# The Role of *Fusobacterium nucleatum* in Colorectal Cancer Cell Proliferation and Migration

**DOI:** 10.3390/cancers14215350

**Published:** 2022-10-30

**Authors:** Zihong Wu, Qiong Ma, Ying Guo, Fengming You

**Affiliations:** 1 Hospital of Chengdu University of Traditional Chinese Medicine, Chengdu 610075, China; 2 TCM Regulating Metabolic Diseases Key Laboratory of Sichuan Province, Hospital of Chengdu University of Traditional Chinese Medicine, Chengdu 610075, China

**Keywords:** colorectal cancer, *Fusobacterium nucleatum*, proliferation and migration, epithelial–mesenchymal transition, tumor microenvironment

## Abstract

**Simple Summary:**

Although the promoting roles of the epithelial–mesenchymal transition (EMT), tumor microenvironment (TME), and oncogenic ncRNAs in tumor metastasis have been fully verified, the mechanisms by which *Fusobacterium nucleatum* (Fn) contributes to the progression of colorectal cancer (CRC) remain poorly understood. The main scope of the current work is to summarize the potential molecular mechanisms by which Fn promotes the proliferation and migration of CRC cells by affecting biological processes, such as EMT, TME, and oncogenic ncRNAs. The ultimate goal is to provide a possible strategy for effective CRC treatment in the near future.

**Abstract:**

Colorectal cancer (CRC) is a common cancer worldwide with poor prognosis. The presence of *Fusobacterium nucleatum* (Fn) in the intestinal mucosa is associated with the progression of CRC. In this review, we explore the mechanisms by which Fn contributes to proliferation and migration of CRC cells from the following four aspects: induction of the epithelial–mesenchymal transition (EMT), regulation of the tumor microenvironment (TME), expression of oncogenic noncoding RNAs, and DNA damage. This review outlines the scientific basis for the use of Fn as a biomarker and therapeutic target in CRC.

## 1. Introduction

Colorectal cancer (CRC) is the third most common malignant tumor worldwide and is associated with high rates of morbidity and mortality [1]. The prognosis of CRC patients is poor, with almost 900,000 deaths annually. The five-year survival rate after distant metastasis is only 8−10% [2]. The incidence of CRC has risen dramatically in China over the past decade [3]. It has been established that the pathogenesis of CRC is complex and diverse, and although the exact mechanisms of its progression and metastasis have not yet been fully elucidated; it is well known that both genetic factors and environmental stimuli play major roles, with data showing that environmental factors contribute to approximately 70% of CRC progression [4].

Metastasis is the major cause of cancer-related deaths. Increasingly more studies are showing that the epithelial–mesenchymal transition (EMT) plays an important role in colorectal cancer progression and metastasis. The EMT is a vital process in which cells lose epithelial characteristics and gain mesenchymal properties, resulting in enhanced cancer cell mobility, invasiveness, and cancer stem-like cell (CSC) properties and the activation of anti-apoptotic pathways, thus conferring cancer cells with their metastatic properties [5,6]. During EMT, cell–cell adhesion and cell–extracellular matrix (ECM) interactions are re-programmed, leading epithelial cell detachment from other cells and the basement membrane, weakening of the epithelial barrier, and the acquisition of mesenchymal properties necessary for migration and invasion [7]. The variability of some adhesion molecules results in distinct modes of collective cancer cell migration, ranging from sheet migration to movement of the cellular cluster [8]. EMT markers may thus serve as potential predictors and targets for CRC [5]. Cancer cells may secrete various cytokines, proinflammatory factor, and chemokines in sculpting the tumor microenvironment (TME) [9]. There is accumulating evidence to indicate that immune cells—the critical ingredient of TME—including macrophages (M), natural killer (NK) cells, and myeloid-derived suppressor cells (MDSCs), take part in tumor progression and metastasis [10]. In recent years, there have been many studies on the correlation between gut microbes and CRC. Local microbiota can reshape the TME and have been recognized as an essential contributor to the risk of developing CRC [11].

*Fusobacterium nucleatum* (Fn) is an oral Gram-negative anaerobe that can cause opportunistic infections. Fn was recently found to be prevalent in human CRC tissues and feces, and it is among the most enriched bacterial proinflammatory species in CRC patients, where it can invade epithelial cells, disrupt signaling, and promote malignant transformation. A significantly increased abundance of Fn in TME is associated with cancer cell proliferation, metastasis, worse prognosis, and chemoresistance [12,13,14]. A recent study confirmed that the oral concentration of Fn is positively correlated with the concentration of colon tissue, and the abundance of Fn could predict CRC prognosis [15]. Existing findings suggest that Fn promotes invasion and migration in CRC by excreting some prominent protein factors, such as *Fusobacterium* adhesin A (FadA) and caspase activation and recruitment domain 3 (CARD3) [16]. Although there is reliable evidence that Fn might be involved in the initiation, progress, and metastasis of CRC, the host-microbe interactions of Fn and its involvement in tumor progression are not fully understood. Therefore, we summarize the mechanisms of Fn that are associated with colorectal cancer cell proliferation and migration from the following aspects: EMT, TME, oncogenic ncRNAs, and DNA damage. Table 1 provides a detailed list of abbreviations.

## 2. Potential Mechanisms by Which Fn May Promote CRC Proliferation and Migration

### 2.1. Proliferative Effects on Primary Tumor

Fusobacteriota, to which Fn belongs, is the dominant phyla of species found in most CRC tissues, patients with a high amount of tissue Fn DNA demonstrate a higher risk for poor outcomes [17]. Fn infection increases the proliferation activities of CRC cell lines and the ability to form xenograft tumors in mice, therefore, the Fn is identified as colorectal cancer “facilitators” [17,18]. A study also finds that FadA promotes SW480 cell growth and proliferation in a dose and time-dependent manner [19]. In addition, Fn-induced miR-4717 promotes CRC cell line proliferation via METTL3-dependent m6A modification [20,21,22]. Lee et al. [23] suggested that Fn might be linked to promoting CRC cell growth, invasion, and pro-tumoral immune responses in MSI-high CRCs. In short, Fn infection has a strong proliferative effect on primary colorectal cancer, which provides a bacterial biomarker and a potential therapeutic target for CRC.

### 2.2. Induction of Epithelial–Mesenchymal Transition

Since the epithelial–mesenchymal transition (EMT) is widely considered to be a major molecular factor driving cancer metastasis, we attempted to identify potential links between EMT and Fn.

#### 2.2.1. E-Cadherin/β-Catenin Complex

E-cadherin is an epithelial marker, and the E-cadherin/β-catenin complex is important for the integrity and adhesion of epithelial cells.

The abundance of β-catenin is higher in Fn-positive CRC tissues than in negative specimens. Fn (ATCC10953/25586 and F01) also increases the levels of β-catenin and its target genes, cyclin D1 and c-Myc, in CRC cells and decreases E-calmodulin concentration, leading to weakened intercellular adhesion as well as abnormal EMT activation. In addition, overexpression of Ki-67, NETO2, and ANGPTL4 has been observed in Fn infection-mouse model. These findings reveal that Fn contributes to promoting the EMT in CRC cells by activating Wnt/β-catenin signaling [3,24,25]. Guo P et al. [26] established a cell-derived xenograft (CDX) mouse model exposed to Fn or FadA knockout (FadA^−/−^ Fn) and found that Fn treatment increased the expression of the cell proliferation markers Ki-67 and PCNA, while FadA^−/−^ Fn decreased the levels of E-cadherin/β-catenin and chk2 in CRC cells. Another study found that Fn infection enhanced the invasion and proliferation of NCM460 cells by interacting with E-cadherin instead of β-catenin without affecting their expression levels. Additionally, Fn was shown to significantly upregulate the levels of NF-κB p65, TNF-α, IL-6, IL-1β, and MMP-13 [27].

Recently, tumor-derived exosomes (TEXs) were reported to be involved in tumor metastasis by transferring carcinogenic miRNA and proteins to normal tissues [28,29]. Fn infection was found to markedly increase exosome secretion from HCT116 and SW480 cells and induce CRC cell migration. The exosomes isolated from Fn-infected HCT116 cells were shown to upregulate the expression of the mesenchymal marker vimentin, increase the levels of cyclin D1 and c-Myc, and downregulate the expression of the epithelial marker E-cadherin via the Wnt/β-catenin pathway. Furthermore, the exosomes isolated from Fn-infected CRC cells were also shown to promote tumor metastasis in vivo, giving the highest number of nodules on the lung surfaces of xenograft mice [29].

Previously, we summarized the main downstream factors involved in the Wnt/β-catenin signaling pathway. Next, we review the upstream factors. Back in 2013, Rubinstein et al. [30] demonstrated that Fn stimulates CRC cell proliferation and regulates inflammation by the activating E-cadherin/β-catenin pathway through its unique FadA adhesin. In 2019, on the basis of previous studies, Rubinstein et al. [17] found that FadA promotes the proliferation and migration of CRC cells in vitro and in vivo by inducing annexin A1, a modulator of Wnt/β-catenin, which increases the levels of cyclin D1 and downregulates E-cadherin. Ternes et al. [31] showed that formate, a metabolite produced by Fn, could increase tumor incidence and expand the Th17 cell population by triggering AhR-Wnt/β-catenin signaling, which led to CRC invasion, metastasis, and CSC. Lu et al. [32] reported that Fn promotes CRC cell metastasis in the liver and lungs of CDX mice by enhancing the translation of EMT-related factors, such as N-cadherin, vimentin, Snail, Zeb1, and Slug while reducing E-cadherin expression via the lncRNA EVADR-YBX1 axis.

In summary, Fn promotes metastasis of CRC mainly through affecting cell–cell adhesion and E-cadherin loss, while the dysregulation of the Wnt/β-catenin signaling pathway is also a typical change during EMT.

#### 2.2.2. Cancer Stem-Like Cells

In addition to the E-cadherin/β-catenin complex, cancer stem-like cells (CSCs) are responsible for cancer progression and the recurrence and metastasis of tumor cells [33,34,35]. Emerging evidence shows that non-CSCs can acquire stem-like features during EMT. The levels of EMT markers (E-cadherin, N-cadherin, and ZO-1) and CSC markers (Nanog, Oct-4, Sox-2, CD44+, and CD133+) is significantly correlated with the abundance of Fn in CRC tissues [35,36,37]. To determine changes in the morphology of CRC cells, Yu et al. [36] cultured HCT8, LOVO, and LS174T cells infected with Fn for 24 h. They found that HCT8 and LOVO cells displayed an elongated shape rather than epithelial cell morphology, and the proliferative activity of cells was promoted. It was further certified that Fn promotes EMT through EGFR/AKT/ERK signaling. Recent observations suggest that Fn infection affects not only colorectal cancer stem-like cells (CCSCs) but also non-CSCs. Fn activates Notch signaling to promote both acquisition of stem-like features by non-CCSCs in addition to CCSC self-renewal via TLR4/MYD88/NF-κB [37].

In brief, these findings indicate that Fn infection increases the stemness of colorectal cancer cells via promotion of the EMT.

#### 2.2.3. Autophagy Signaling

Autophagy is a highly conserved catabolic process that is referred to as a “double-edged sword” in tumors, as it can promote or suppress cancer development. Evidence has confirmed that activation of autophagy can serve to strengthen or suppress the EMT, contributing to metastasis by regulating various signaling pathways [34,38], and it is also one of the mechanisms by which Fn promotes chemoresistance of CRC [39]. In a xenograft mice model and SW480/HCT116 cells, Fn(F01) induces CRC cell metastasis by activating the autophagy pathway via increasing the levels of CARD3, is a premetastatic kinase. This is accompanied by declines in E-cadherin and p62 and a rise in vimentin. Furthermore, chronic Fn infection stimulates tumor growth and promotes metastasis (e.g., lung, liver, and lymph nodes) in APC^Min/+^ mice, leading to aberrant activation of autophagic signaling pathways and synergistic oncogenic effects [38].

**Figure 1:**
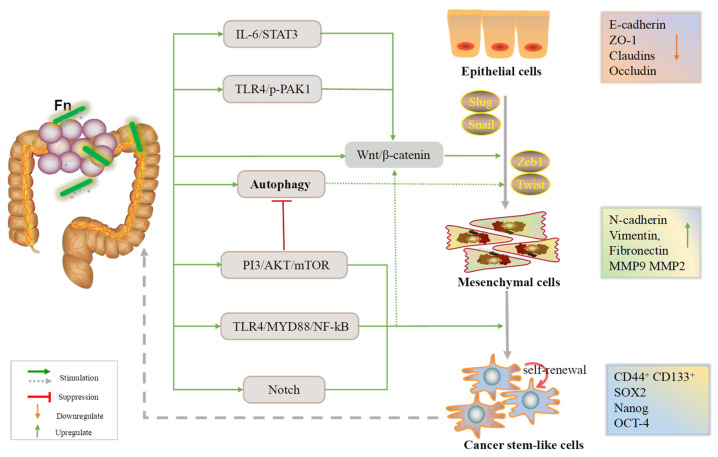
An overview of the EMT process during which Fn is involved in the development of CRC.

In a nutshell, Fn infection can affect cell–cell adhesion, contributing to the EMT process by influencing CRC cell proliferation, invasion, and migration. As mentioned, the E-cadherin/β-catenin complex, CSCs, and autophagy signaling are the three main axes by which Fn promotes the EMT (Figure 1).

### 2.3. Reshaping the Tumor Microenvironment

The Tumor Microenvironment (TME), including the tumor cells, noncancerous cells, and surrounding stroma, is crucial for malignant progression. Fn infection can contribute to CRC proliferation and migration by reshaping the TME.

#### 2.3.1. Tumor-Infiltrating Immune Cells

Fn infection can recruit tumor-infiltrating immune cells, in particular myeloid-derived suppressor cells (MDSCs), tumor-associated macrophages (TAMs), tumor-associated neutrophils (TANs), and dendritic cells (DCs), thereby suppressing potent immunity in CRC [40,41,42].

MDSCs have become an important part of the TME and have potent immune suppressive activity [43]. These cells are facilitated by cancer cells and stromal cells in the TME, and an expanded MDSC population has been shown to suppress the anti-tumor immune response and promote angiogenesis [44]. Kostic et al. [40] observed an increase in CD11b+ myeloid cells in the cancers of APC^Min/+^ mice fed Fn. MDSCs were enriched by a mean of 3.7× in Fn fed mice compared with controls. Additionally, MDSCs could significantly suppress T cell activity. It was found that both TANs and TAMs were enriched (mean of 13.4× and 7.8× increases in the cell number, respectively) in Fn fed mice. They also suggested an NF-κB-driven pro-inflammatory response for the upregulation of COX-2, TNF-α, IL6, IL1α or β, IL8, and MMP3 levels. Through testing the hypothesis that Fn infection might be associated with lower T cell abundance in colorectal cancer liver metastases (CRLM), Sakamoto et al. [41] showed that Fn-CRLM is associated with a significantly higher density of MDSCs (CD33+) and TAMs (CD163+) and a lower density of CD8+ T cells.

Macrophages (M) can polarize to the M1 or M2 phenotypes due to their high plasticity. Influenced by specific environmental factors, macrophages are probably transformed to the M2 phenotype when reprogramming the TME [45]. Evidence has found that Fn promotes M2 macrophage polarization through activation of the NF-κB pathway, allowing it to participate in TME reprograming and stimulating the metastasis of CRC in vitro and in vivo [46,47]. Meanwhile, Fn infection significantly increases the proportion of M2-phenotype macrophages (CD206+, CD68+, CCR6+, F4/80+) while decreasing the proportion of M1-phenotype macrophages (CD86+, iNOS) [42,46,47]. Data show that macrophages are major tumor-infiltrating immune cells in human Fn-CRC. Fn infection increases M2 polarization and enhances intestinal tumor cell proliferation through the TLR4/IL-6/p-STAT3/c-Myc cascade [42]. Lamprinaki et al. [48] observed that the U-937 cell line, when stimulated by Fn, acquires an M2-phenotype. In addition, Fn (ATCC 51191) and its derived components (OMVs, LPS) might induce IL-10 and IL-8 production while downregulating CD86+.

In summary, Fn infection can recruit tumor-infiltrating immune cells, suppressing the potent immune activity of CRC cells. Thus, a favorable immune microenvironment is created for tumor cell proliferation.

#### 2.3.2. Prometastatic Cytokines

Proinflammatory cytokines, chemokines, and adhesion molecules are important extracellular matrix (ECM) components found in the tumor microenvironment. FadA can induce chemokine IL-8 and CXCL1 secretion in HCT116 cells, which promotes cancer cell migration [49]. Chemokine IL-8, CXCL16, and RhoA were also found to be more enriched in exosomes isolated from Fn-infected HCT116 cells than from uninfected HCT116 cells [29]. More importantly, circulating exosome CXCL16 levels are positively associated with Fn abundance and distant metastasis in patients with CRC. The liver is commonly the site corresponding to distant metastasis of CRC. To investigate the role of Fn in CRC liver metastasis, CT26-Luc mice were given Fn orally, and the results show a significant increase in the rate of liver metastasis in the mouse model. Additionally, the serum levels of IL12, IL6, IL17A, CXCL1, IFN-γ, TNF-α, and other proinflammatory cytokines in these mice were substantially increased [50]. The key step in the tumor metastatic process is that cancer cells adhere to endothelial cells for extravasation. Zhang et al. [51] discovered that Fn could promote CRC cell adherence to endothelial cells, extravasation, and migration by activating NF-κB signaling, and the cell surface adhesion molecule ICAM1 was also shown to be upregulated due to the phosphorylation of p65 subunit of NF-κB.

All of these prometastatic cytokines induced by Fn infection contribute to reprogramming of the tumor microenvironment.

#### 2.3.3. Tumor Metabolism

One of the most striking characteristics of tumor cells is their ability to adapt to changing environmental conditions by utilizing a wide range of nutrients to maintain their demanding anabolic demands [52]. With the exception of various tumor-infiltrating immune cells and cytokines, tumor metabolism, including amino acid metabolism [53] and glycolysis [54,55], also play important roles in CRC invasion and metastasis. Fn can regulate amino acid metabolism by intervening in the distribution of the intestinal microbiota. In SW480 cells exposed to Fn, the levels of lactic acid, aspartic acid, and anti-apoptotic protein Bcl-2 are increased, whereas concentrations of propionic acid and the apoptotic protein Bax are decreased; in the tumor tissues and serum of CRC patients, the levels of isoleucine and leucine are decreased. These findings in vivo/in vitro suggest that Fn inhibits cancer cell apoptosis, promoting CRC progression, mainly by regulating propionic acid and lactic acid metabolism [53]. The enrichment and persistence of Fn bacterial community in CRC is essential for tumor progression. Fn infection has been experimentally demonstrated to promote glycolysis by targeting ANGPTL4 and lncRNA ENO1- IT1, which facilitates Fn colonization of the intestinal mucosa [54,55]. In line with the Warburg effect, glycolysis is more conducive to glucose catabolism and constitutes a major driver of tumor progression [56]. Enhanced glycolysis further promotes tumor cell proliferation and aids in the maintenance of a hypoxic microenvironment, which creates unique inhabitant niches and ensures the persistence of anaerobic bacteria.

Taken together, these abnormal metabolic processes sustain tumor cells energy production rates and contribute to the malignant transformation of intestinal epithelial cells.

#### 2.3.4. Tumor-Associated Microbiota

The local microbiota is also an important part of the tumor microenvironment. Oncogenic microbial environment can selectively allow certain microorganisms to survive and make it favorable for tumor growth. The interactions between various microbial species have been shown to influence CRC progression. Fn infection alters the structure of the colonic mucosal microbial community. The significant enrichment of potentially pathogenic taxa (e.g., phylum *Proteobacteria*, *Stenotrophomonas*, *Denitrifaciens*, and unidentified *Enterobacteriaceae*) and depletion of probiotic bacteria (such as *Lactobacillus*) are features of the mucosal microbiota caused by Fn infection, which further worsening the dysbiosis of the colonic mucosal microbiota [3]. Yu et al. [53] collected feces of mice infected with Fn, and they foud that the abundance of Fn was positively correlated with the abundance of *Gemmatimonadetes* while negatively correlated with the abundance of *Tenericutes* and *Euryarchaeota*. Bullman et al. [57] confirmed that the relative abundance of the overall dominant microbiome (e.g., *Bacterium*, *Prevotella* and *Selenomonas* species), including Fn, was nearly identical in the primary CRC and their matched metastatic cancers. These findings suggest that Fn might migrate together with the CRC cells to the metastatic site and be persistently associated with distant metastases. Another study found that *Fusobacterium nucleatum*, *Prevotella*, and *Dialister* were enriched in metastatic CRC tissues [38]. However, extensive research into bacteria–tumor cell interactions and metabolic crosstalk is required.

**Figure 2:**
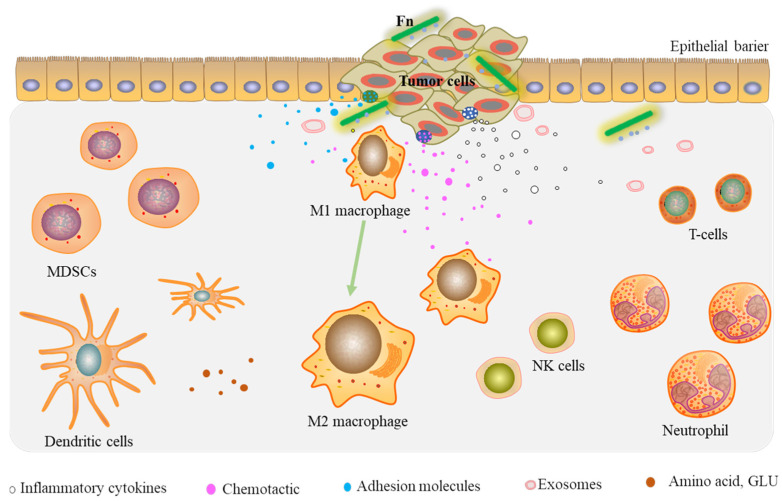
Fn reprograms the TME by recruiting tumor-infiltrating immune cells and prometastatic cytokines.

Overall, these findings support the idea that Fn infection can reprogram the tumor microenvironment of CRC by recruiting tumor-infiltrating immune cells, inducing the secretion of prometastatic cytokines, modulating tumor metabolism, and enriching the tumor-associated microbiota, leading to tumor cell growth and metastasis (Figure 2).

### 2.4. Oncogenic ncRNAs

#### 2.4.1. LncRNAs

There is increasing evidence that long noncoding RNAs (lncRNAs) are widely associated with tumor proliferation and metastasis [32,58,59]. Lu et al. [32] found that the lncRNA EVADR is specifically upregulated in Fn-infected metastatic CRC tissue through YBX1-dependent translation, which might be a promising finding for research on the prevention of CRC metastasis. Data reveal that the abundance of Fn is significantly upregulated in CRC patients with lymph nodes and lung metastasis [60]. Mechanically, Fn infection upregulates the expression of lncRNA KRT7-AS and KRT7 in HCT-116 and LoVo cells by activating NF-κB signaling. Consistent with this, knockdown of KRT7-AS significantly results in a significant improvement in the occurrence of lung metastases in mouse model [60]. Furthermore, Fn can alter the biological function of CRC cells by activating lncRNA ENO1-IT1 transcription which, in turn, influences the histone modification pattern on KAT7 target genes [55].

#### 2.4.2. miRNAs

MicroRNAs (miRNAs) are small ncRNAs. Numerous miRNAs have been found to be upregulated or downregulated in human tumors. The dysfunction of miRNAs in turn disturbs the expression of either tumor promoter or tumor suppressor [61,62]. Guo S et al. [29] suggested that exosome miRNAs exhibit a variety of biological functions during Fn infection. For example, exosomes could deliver miR-1246/92b-3p/27a-3p from Fn-infected cells into uninfected cells to promote migration behaviors, and the level of GSK3β, the target gene of the oncogenic miRNAs, was found to be significantly reduced in exosomes from Fn infection CRC cells. A study characterizing several miRNAs and genes associated with the development of Fn-induced CRC found that approximately 121 miRNAs were aberrantly expressed in CRC tissue following Fn infection. Among these miRNAs, the expression levels of miR-4474 and miR4717 were significantly increased, while the target gene, CREBBP, was decreased in CRC tissue exposed to Fn [20,21]. Fn infection also enhances the tumor formation ability of xenografts and increases the levels of inflammatory factors in mouse model [18]. Approximately 50 miRNAs are found to be significantly upregulated in CDX mice, and the level of miR21 increases by more than four-fold. Furthermore, Fn infection not only induces the expression of miR21 but also reduces the level of RASA1 via the TLR4/MYD88/NF-κB signaling pathway [18]. In addition, some important miRNAs are lost in CRC tissues after Fn interference. Xu et al. [46] found that chemokine CCL20 tended to increase in patients with stage IV metastatic CRC. Using luciferase reporter experiments, they also found that miR-1322 could directly bind to CCL20, and its expression was found to be negatively associated with Fn abundance and CCL20 levels. These findings indicate that Fn infection promotes CRC metastasis via the miR-1322/CCL20 axis. FOXD3 is a transcription factor for METTL3, an m6A methyltransferase, and there is evidence that Fn could diminish the expression of FOXD3 and suppress the transcription of METTL3 via YAP pathway activation. Its target, KIF26B, is promoted by the decline in m6A modifications and mRNA degradation, which are responsible for Fn-related CRC metastasis [22].

In summary, the malfunction of oncogenic ncRNAs and their target genes are key factors in the promotion of colorectal cancer progression and metastasis.

### 2.5. DNA Damage

DNA damage is an important first step in the process of carcinogenesis, which is associated with genomic instability and chronic inflammation. Fn infection has been shown to promote DNA damage and induce the malignant transformation of cells [63,64]. Using Fn in a co-culture with EDMs, Sayed et al. [64] found that the expression of NEIL2 (a DNA glycosylase) was significantly suppressed among the key DNA repair proteins, while the levels of inflammatory cytokines and double-strand breaks in EDM cells were higher with NEIL2-null, which might favor the development of CRC. Based on a previous study, Guo P et al. [26] also established a C57BL/6J-adenomatous polyposis coli (APC) Min/J (APC^Min/+^) mouse model and confirmed that the levels of γH2AX (a DNA double-strand breaks marker) and chk2 were significantly higher in CRC tissues from Fn infected mice, which led to DNA damage and tumor growth as well as increased numbers of CRC cells in the S phase of the cell cycle.

Collectively, these findings suggest that Fn can promote CRC proliferation and migration by interfering with a variety of biological processes, such as induction of the epithelial–mesenchymal transition, reprogramming of the tumor microenvironment, expression of oncogenic ncRNAs, and disruption of host DNA. The detailed mechanisms by which *Fusobacterium nucleatum* acts in CRC models are shown in Table 2.

## 3. Discussion

### 3.1. Current Status

Here, we provide a review of potential mechanisms by which *Fusobacterium nucleatum* might promote colorectal cancer cell proliferation and migration. Existing studies show that Fn might contribute to the proliferation and metastasis of CRC by affecting a variety of mechanisms, including the induction of the EMT, the regulation of the TME, the expression of oncogenic ncRNAs, and DNA damage.

**Figure 3:**
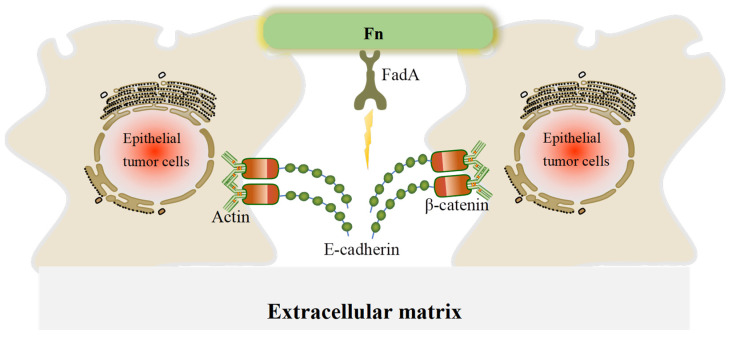
A schematic of the effect of FadA in disrupting adherence junctions in CRC cells. In adherence junctions, E-cadherin and β-catenin form complexes on epithelial cell membranes and bind to the actin cytoskeleton to maintain cell–cell adhesion. When FadA invades, it can bind to E-cadherin, resulting in its separation from β-catenin and subsequent activation of β-catenin signaling, leading to a reduction in cell–cell adherence between cancer cells.

Fn is a ubiquitous opportunistic pathogen that plays an emerging role in CRC and other tumors. CRC patients with high abundance of Fn have poor prognosis and are more likely to develop metastases [16,35]. Fn can interact with host cells and mediate important biofilm-organizing behavior by excreting numerous adhesins. For example, fimbriae and nonfimbrial adhesins help Fn to attach to other bacteria and cells, which contributes to Fn colonization [12]. FadA and Fap2 are virulence factors in Fn and the most common fimbrial adhesin proteins. They function to dissociate the adherence junctions between host cells, leading to tumor cell migration and invasion [65,66]. The EMT is a process during which epithelial cells lose cell–cell and cell–extracellular matrix adhesion and acquire mesenchymal cell phenotypes. In our review, the disassembly of the E-cadherin/β-catenin complex is the main EMT mechanism by which Fn promotes CRC metastasis, E-cadherin mediates Fn adhesion to epithelial cells, and β-catenin regulates cell growth and cell–cell adhesion in the maintenance of epithelial cell layers (Figure 3). Fn also promotes translocation of β-catenin to the nucleus, thereby activating the transcription factor Wnt and triggering downstream signaling.

CSCs are precursors of metastasis and have self-renewal and differentiation abilities. They can also allow the tumor to clonally reproduce itself over the long term [67]. In these findings, non colorectal cancer stem-like cells (CCSCs) infected with Fn are able to acquire stem-like features under the influence of the EMT process (Figure 1), and cancer stem-like markers have been found in metastatic CRC. Recent studies have reported that autophagy, a highly conserved catabolic process, plays dual roles in tumor initiation and development, thus allowing cancer cells to survive when subjected to environmental stresses [34]. There is a complex relationship between EMT-related signaling and autophagic pathway. For instance, activation of the PI3K/AKT/mTOR signaling not only inhibits the autophagic signaling but also reduces the expression of E-cadherin [68,69]. It is consistent with the conclusion reached in this paper that Fn can activate autophagic signaling and regulate the expression of EMT markers.

Fn infection also establishes an appropriate microenvironment to promote colorectal cancer cells growth and proliferation. Including through the recruitment of tumor-infiltrating immune cells, overexpression of prometastatic cytokines, and regulation of tumor metabolism. Thanks to a microenvironment suitable for survival, CRC cells tend to collect Fn more frequently. Under these pathogenic conditions, Fn affects the function of host immune cells, leading to immune evasion of tumor cells. Overall, tumor-infiltrating immune cells, pro-metastatic cytokines, tumor metabolism, and interacting microflora provide important microenvironmental conditions for Fn to promote colorectal cancer cells proliferation and migration.

**Figure 4:**
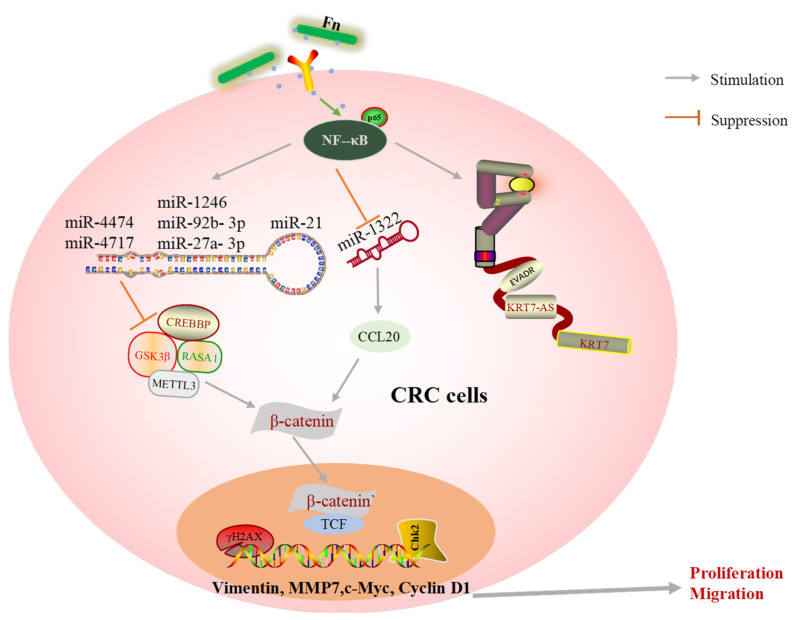
A schematic of oncogene expression regulated by Fn in CRC metastasis.

Noncoding RNAs (ncRNAs) have been identified as both oncogenic promoters and cancer suppressors in a variety of cancers [70]. According to our review and previous findings, it is likely that ncRNAs may be central to the molecular mechanism by which Fn induces tumor cell migration and metastasis (Figure 4). LncRNAs and miRNAs are the main ncRNAs mentioned in this review. miRNAs have emerged as major participants in the interactions between host cells and pathogens. There are a wealth of studies confirming that Fn infection leads to significant changes in miRNA. These findings suggest that Fn promotes CRC development by upregulating or downregulating oncogenic ncRNA. DNA damage induced by chemicals, physical agents, and other harmful substances can be repaired by special DNA repair tools. However, damaged DNA may have replicated before it is repaired, leading to gene mutations and limitless replicative potential [71]. Imperfect DNA repair results in genomic instability and affects the fate of downstream cells. The accumulation of mutations in a cell’s DNA results in tumorigenesis [72]. Fn infection reduces the level of the key DNA repair protein, abolishes the downstream repair process and results in the accumulation of γH2AX, a DNA double-strand break marker, and excessive replication of abnormal DNA (Figure 4).

**Figure 5:**
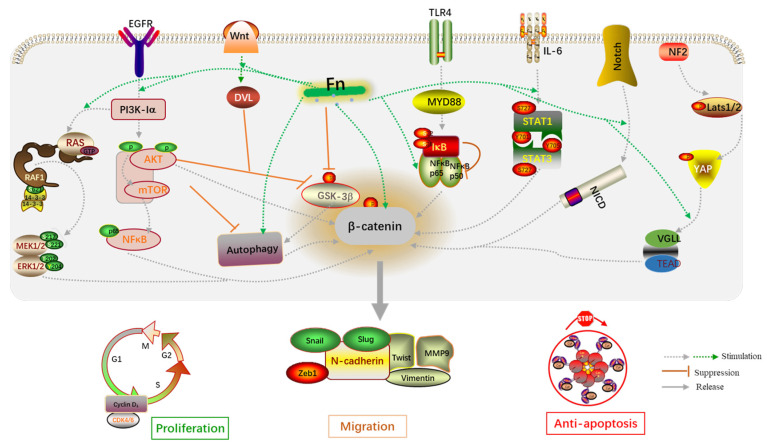
An overview of the molecular mechanisms by which Fn is associated with CRC proliferation and migration.

In addition, the biological behaviors of host cells induced by Fn infection are transmitted through signaling pathways. Wnt/β-catenin, TLR4/NF-κB, IL-6/STAT3, autophagy, AKT/ERK, CXCL16/IL-8, and Hippo-YAP are classical signaling pathways derived from Fn infection and included in this review (Figure 5). The dysregulation of Wnt/β-catenin signaling has been demonstrated to drive cancer cell proliferation, invasion, and migration. For example, the Wnt/β-catenin pathway can be activated by miR-454-3p-mediated suppression, thereby promoting breast cancer metastasis [73]. RUNX1 promotes CRC metastasis through the induction of Wnt/β-catenin signaling and the EMT [74], while cinobufacini can inhibit CRC invasion and metastasis by inhibiting the Wnt/β-catenin–EMT pathway [75]. NF-κB has been widely implicated in the angiogenesis, proliferation, and metastasis in CRC, owing to crosstalk with other signaling pathways. B7-H3 and SREBP1 can increase angiogenesis of endothelial cells by promoting the phosphorylation of NF-κB p65 [76,77]. Additionally, upregulation of MMP7 driven by NF-κB promotes the invasion and metastasis of CRC cells [77]. Experiments have shown that cancer-associated fibroblasts upregulate the inflammatory protein LRG1 as well as inducing the EMT to promote CRC cell invasion and migration through the IL-6-STAT3 axis [78]. The IL-6/STAT3 pathway also affects the tumor-infiltrating immune cells in the TME in CRC and protects cancer cells from apoptosis [79]. In summary, the abnormal activation of these classical signaling pathways is indeed a key mechanism by which Fn infection promotes CRC cell proliferation and metastasis.

### 3.2. Future Directions

Given the specificity of this anaerobic bacterium in colorectal cancer and the feasibility of measurement, *Fusobacterium nucleatum* is expected to serve as a diagnosis biomarker for CRC in future. However, there are still some limitations.

Firstly, to date, mainly animal and tumor cells models have been used to explore the interaction between the microbiota and host in CRC progression, but the results must be taken with caution when referring to human CRCs; that is, more organoid models are needed to research the mechanisms by which the microbiota promote CRC progression and migration. Recently, a three-dimensional organoid culture system derived from human tumor issue was developed [80,81]. Based on the culture techniques used, colorectal cancer-derived organoids have now been successfully established and are widely used, which may allow overcoming many previous limitations [82,83,84]. Wang et al. [85] successfully established a panel of organoid cultures derived from multiple types of human CRC specimens. They found that organoids expressed typical intestinal epithelial cell markers and the ratio of stem cell-like cells was increased. Additionally, Wang et al. [85] confirmed that organoids in culture could accurately reflect in vivo gene expression patterns. These findings confirm the use of organoid culture systems to explore the Fn association with CRC progression with a high degree of confidence, and the organoids derived from human tumor tissues are a promising future human model alternative.

Secondly, reliable and rapid measurement methods to validate the feasibility of Fn as a biomarker are lacking. Considering that Fn may originate from the oral cavity and that saliva is readily available through non-invasive routes, Zhang et al. [86] reported that salivary Fn DNA could be used as a biomarker for colorectal cancer diagnosis and prognosis. In their research, they discovered that CRC patients with regional lymph node metastasis and distant metastasis had increasing levels of salivary Fn DNA (median: 4.662 IQR, 5.856 IQR, respectively). The sensitivity and specificity levels for CRC in the test cohort were 86.7% and 67.2%, respectively, when the cutoff value was 0.437. Zhang et al. [86] also identified a total of 1287 differentially expressed mRNAs based on different Fn DNA concentrations. Measuring the levels of salivary Fn DNA and related mRNAs appears to be more sensitive and suitable model than in vitro experiments and animal models. For example, significant differences were found in 91 types of miRNA depending on whether they were in exosomes isolated from Fn-infected or uninfected HCT116 cells [29]. In a xenograft animal model, 50 miRNAs were found to be significantly upregulated, whereas 52 miRNAs were significantly downregulated in CDX mice [18]. In short, the detection of salivary Fn DNA has certain advantages compared with existing detection methods.

Finally, except for known virulence factors, such as FadA, Fap2, LPS, and OMVs, other specific metabolites from Fn still need to be further investigated. Moreover, it is unclear whether Fn is completely harmful in the process of tumor progression needs to be further investigated, and objective interpretation of experimental data needs to be carried out.

## 4. Conclusions

In our review, we addressed the diverse roles of Fn during CRC cell proliferation and migration. The potential mechanisms mainly include the induction of EMT, regulation of the TME, expression of oncogenic ncRNAs, and promotion of DNA damage. Collectively, Fn promotes the proliferation and metastasis of CRC, mainly by affecting cell–cell adhesion, reshaping the extracellular microenvironment, regulating oncogene expression, and activating prometastatic signaling pathways. In addition, Fn is expected to serve as a diagnosis biomarker as well as a potential therapeutic target for CRC. While these findings are promising, there are still some limitations to the current work. These conclusions need to be further validated in organoid models as well as in clinical practice. Furthermore, easy-to-operate and reliable methods for detecting the abundance of Fn should also be developed in the future.

## Figures and Tables

**Table 1:** Abbreviations used in this article.

Abbreviations	Full Name	Abbreviations	Full Name
APC	adenomatous polyposis coli	lncRNAs	long noncoding RNAs
ATCC	American Type Culture Collection	MDSCs	myeloid-derived suppressor cells
CCSCs	colorectal cancer stem-like cells	miRNAs	microRNAs (small ncRNAs)
CDX	cell-derived xenograft	ncRNAs	noncoding RNAs
CSCs	cancer stem-like cells	PDX	patient-derived xenograft
ECM	extracellular matrix	TAMs	tumor-associated macrophages
EMT	epithelial–mesenchymal transition	TANs	tumor-associated neutrophils
FadA	*Fusobacterium nucleatum* adhesin A	TEXs	tumor-derived exosomes
Fn	*Fusobacterium nucleatum*	TME	tumor microenvironment

**Table 2:** Potential mechanisms of *Fusobacterium nucleatum* in CRC models.

Mechanisms	References	Fn Strain	Models	Findings			Experiment	Metastatic Sites
				Signaling	Upregulates	Downregulates		
Induced EMT								
E-cadherin/β-catenin	[24]	F01 ATCC 10953	CRC tissue SW480, Caco-2	Activates β-catenin signaling via a TLR4/p-PAK1/β-catenin S675 cascade	β-catenin TLR4 PAK1 c-Myc cyclin D1		In vitro	
	[25]	ATCC 25586	CRC tissue DLD-1, SW480	Activates Wnt/β-catenin and IL-6/STAT3 signaling via upregulation of Cdk5	CDK5 β-catenin c-Myc cyclin D1 p-STAT3 IL-6,8 COX-2 TNF-β		In vitro	
	[3]	ATCC 25586	AOM/DSS mice	Impairs the function of the intestinal barrier and the aberrant activation of EMT	Ki-67 NETO2 ANGPTL4 PCK1	E-cadherin	In vivo	
	[26]	ATCC 25586 (FadA)	CRC tissue HCT29, HT116 CDX mice, APC^Min/+^ mice	Activates the E-cadherin/β-catenin pathway, leading to the upregulation of chk2	chk2 β-catenin Ki-67 PCNA	E-cadherin	In vitro In vivo	
	[27]	ATCC 25586	NCM460	Interacts with E-cadherin and enhances the malignant phenotype of CRC cells	NF-κB p65 IL-6 IL-1β MMP-13 TNF-α		In vitro	
	[29]	Fn-Ex (TEXs)	HCT116, SW480 CDX mice	Activates the Wnt/β-catenin pathway	β-catenin vimentin c-Myc cyclin D1	E-cadherin	In vitro In vivo	Liver lung
	[30]	ATCC 12230 US1U SF81 (FadA)	CRC tissue HCT116, DLD1, HT29, SW480, RKO CDX mice	Regulates the inflammatory and oncogenic responses via E-cadherin/β-catenin	β-catenin NF-κB IL-6,8,18 LEF/TCF c-Myc cyclin D1	E-cadherin	In vitro In vivo	
	[17]	WAL12230 ATCC 25586 (FadA)	CRC tissue 10C, HCT116, DLD1, RKO, SW480, HT29 CDX mice, APC^Min/+^ mice	Induces the Wnt/β-catenin modulator Annexin A1	Annexin A1 β-catenin cyclin D1	E-cadherin	In vitro In vivo	
	[31]	ATCC 23726 ATCC 25586 (formate)	CRC tissue HCT116, Caco-2 CDX mice	Induces cancer stemness and thereby metastatic dissemination by activation of the AhR-Wnt/β-catenin pathway	AhR β-catenin IL8 Th17 MAPK ERK MEK-p38 NF-κB p65		In vitro In vivo	lung
	[32]		CRC tissue HCT116, LoVo, SW480, SW620, HT29 CDX mice	Enhances the translation of EMT-related factors by the lncRNA EVADR-YBX1 axis	N-cadherin vimentin Snail Slug Zeb1	E-cadherin	In vitro In vivo	liver lung
CSCs	[35]		CRC tissue	Potential involvement of Fn in EMT–CSC crosstalk	N-cadherin Nanog Oct-4 Sox-2	E-cadherin	In vitro	lung, liver ovary node
	[36]		LOVO, HCT8, LS174T AOM/DSS mice	Promotes the EMT through EGFR/AKT/ERK signaling and increases the stemness of CRC cells	Fibronectin N-cadherin Snail Slug CD44+ CD133+ IL-1β, 6 p-EGFR p-AKT p-ERK	E-cadherin ZO-1	In vitro In vivo	
	[37]	ATCC 25586	CRC tissue CCSCs, HEK293, HT29, HCT116 CDX mice	Promotes Numb degradation and activates Notch signaling in non-CCSCs; enhances CPT1B-mediated FAO via the TLR4/MYD88/NF-κB in CCSCs	ALDH1 CD133+ CD44+ Lgr5 Olfm4 Sox9 Aldh1 FASN NICD MDM2 CPT1B NF-κB	Numb	In vitro In vivo	
Autophagy	[38]	F01 ATCC10953	CRC tissue SW480, HCT116, CT26 xenograft mice APC^Min/+^ mice	Activates autophagy signaling by upregulating CARD3	CARD3 LC3-II beclin1 vimentin ATG5,7 MAPK	E-cadherin p62	In vitro In vivo	Lung liver node
Reprogramed TME								
MDSCs	[40]	EAVG_002	CRC tissue APC^Min/+^ mice	Generates a proinflammatory microenvironment through the recruitment of tumor-infiltrating immune cells	MDSCs (CD11b^+^) TANs, TAMs DCs COX-2 IL1,6,8, TNF MMP3 NF-κB p65		In vitro In vivo	
	[41]		CRC tissue APC^Min/+^ mice	The presence of Fn is associated with a lower density of CD8+ T cells and a higher density of MDSCs in CRC liver metastases	MDSCs (CD33^+^) TAMs (CD163^+^) Ki67 TIL- 6 TNF-α	CD8^+^	In vitro In vivo	liver
M2 polarization	[46]	ATCC 25586	CRC tissue HCT116, LoVo CDX mice	Promotes M2 polarization through the NF-κB pathway	F4/80^+^ CCR6^+^ CD206^+^ ARG1 MRC1 IL-10 TGF-β		In vitro In vivo	lung
	[47]	ATCC 25586	CRC tissue HCT116, SW480, THP-1 CDX mice	Promotes M2 polarization via the TLR4/NF-κB/S100A9 pathway	CD206^+^ IL-10 S100A9 TLR4 NF-κB p65 PCNA N-cadherin VEGF TGF-β	CD86+ iNOS TNF-α	In vitro In vivo	
	[42]	ATCC10953 F01	CRC tissue RAW 264.7 APC^Min/+^ mice	Promotes the M2 polarization of macrophages via the TLR4/IL-6/p-STAT3/c-Myc cascade	CD206^+^ IL-10 TGF-β TLR4 IL-6 p-STAT3 c-Myc	CD86^+^ IL-12 TGF-α	In vitro In vivo	
	[48]	ATCC 51,191 (LPS/OMVs)	CHO-Siglec-7-Fc, U937, moDCs, moM¢s	Promotes M2 polarization and a proinflammatory environment via the Fn–Siglec-7 interaction	Siglec-7 IL-8 I-10 IL-10 PD-L1	CD86^+^ TNF-α	In vitro	
Prometastatic cytokines	[49]	ATCC 23,726 (FadA, Fap2)	HCT116	Induces the secretion of the proinflammatory and prometastatic cytokines	IL-8 CXCL1		In vitro	
	[29]	Fn-Ex (TEXs)	HCT116, SW480 CDX mice	stimulates the CXCL16/RhoA/IL-8 exosomes	IL-8 CXCL16 RhoA	CXCR6	In vitro In vivo	Liver lung
	[50]	ATCC 25586	CT26-Luc mice	Affects the secretion of inflammatory cytokines and modulates the hepatic immune response	IL-6,12, 9 IL-17A CXCL1 MCP-1 TNF-α IFN-γ CD11b^+^ Treg	CD3^+^ CD4^+^ CD8^+^ NK Th17	In vivo	liver
	[51]	ATCC 25586 ATCC 10953	CRC tissue HCT116, LoVo, HUVECs CDX/PDX mice	Promotes CRC cell adhesion to endothelial cells and metastasis by activating the NF-κB/ICAM1 axis	ICAM1 ALPK1 NF-κB p65		In vitro In vivo	Liver Lung node
Tumor metabolism	[53]	ATCC 25586	CRC tissue SW480 AOM mice	Regulates amino acid metabolism	lactic acid aspartic acid glutamate glutathione Bcl-2 caspase 3	propionic acid leucine isoleucine Bax	In vitro In vivo	
	[54]	ATCC 25586	CRC tissue DLD1, SW480, HT-29, HCT-116 CDX mice	Promotes glycolysis via the induction of ANGPTL4 and facilitates the colonization of Fn	ANGPTL4 H3K27ac ECAR GLUT1	HDAC	In vitro In vivo	
	[55]		CRC tissue HCT116, DLD1 CDX mice	Promotes CRC glycolysis via ENO1	lactic acid ECAR		In vitro In vivo	
	[3]	ATCC 25586	AOM/DSS mice	Alters the colon mucosal microbiota	FABP1 VAV2 GalR1	E-cadherin	In vivo	
Oncogenic ncRNAs								
lncRNAs	[32]		CRC tissue HCT116, LoVo, HT29, SW480, SW620 CDX mice	Regulates the lncRNA EVADR-YBX1 axis	EVADR YBX1		In vitro In vivo	Liver lung
	[60]		CRC tissue HCT116, LoVo CDX mice	Upregulates KRT7-AS/KRT7 by activating the NF-κB pathway	KRT7-AS KRT7 NF-κB p65	IκB-α	In vitro In vivo	lung
	[55]		CRC tissue HCT116, DLD1 CDX mice	Targets lncRNA ENO1- IT1 to promote glycolysis and oncogenesis	ENO1-IT1 ENO1 KRT7		In vitro In vivo	
miRNAs	[29]	Fn-Ex (TEXs)	HCT116, SW480 CDX mice	Stimulates tumor cells to generate miR-1246/92b- 3p/27a- 3p- rich	miR-1246 miR-92b-3p miR-27a-3p	GSK3β	In vitro In vivo	Liver lung
	[21]		CRC tissue Caco-2, HEK-293	Upregulates miR-4474/4717 by post transcriptionally regulating the target gene, CREBBP	miR-4474 miR-4717 STAT1 TP53 EWSR1	CREBBP JUN PRKACB CAMK2B	In vitro	
	[18]		CRC tissue HCT116, HT29, LOVO, SW480 CDX mice, APC^Min/+^ mice	Regulates miR21 expression through the TLR4/MYD88/NF-κB pathway	miR-21 IL-17,21,22 MIP3a TLR4,2 MYD88 NF-κB p65 MAPK	RASA1 PDCD4 PTEN RECK SPRY1 RHOB IκB-α	In vitro In vivo	
	[22]	ATCC 25586	CRC tissue HCT116, LoVo, RKO, SW620 PDX mice	Reduces m^6^A modifications through the Hippo-YAP/FOXD3/METTL3/KIF26B axis	KIF26B YAP NF-κB	FOXD3 METTL3 m6A NF2 KIBRA Willin\FRMD6	In vitro In vivo	Liver bone
	[46]	ATCC 25586	CRC tissue HCT116, LoVo CDX mice	Regulates miR-1322/CCL20 expression through the NF-κB pathway	CCL20	miR-1322	In vitro In vivo	lung
DNA damage								
	[64]	ATCC 25586	CRC tissue EDMs APC ^Min/+^ mice	Induces the downregulation of NEIL2 and the consequent accumulation of DNA damage	NTH1 MSH2 MSH6 Ku70 IL-8 pATM p-γH2AX	NEIL2 NEIL1 PMS2	In vitro In vivo	
	[26]	ATCC 25586 (FadA)	CRC tissue HCT29, HT116 CDX mice, APC ^Min/+^ mice	Elevates DNA damage and increases the number of CRC cells in the S phase of the cell cycle	chk2 γH2AX		In vitro In vivo	

Note: Fn, *Fusobacterium nucleatum*; FadA, *Fusobacterium nucleatum* adhesin A; EMT, epithelial–mesenchymal transition; TME, tumor microenvironment; ncRNAs, noncoding RNAs; lncRNA; long noncoding RNA; miRNA, microRNA (small ncRNA); MDSCs, myeloid-derived suppressor cells; ATCC, American Type Culture Collection; CDX, cell-derived xenograft; PDX, patient-derived xenograft; CSCs, cancer stem-like cells; APC, adenomatous polyposis coli.
